# Rendered invisible? The absent presence of egg providers in U.K. debates on the acceptability of research and therapy for mitochondrial disease

**DOI:** 10.1007/s40592-015-0046-7

**Published:** 2015-12-28

**Authors:** Erica Haimes, Ken Taylor

**Affiliations:** Policy, Ethics and Life Sciences (PEALS) Research Centre, Newcastle University, 4th Floor Claremont Bridge, Claremont Road, Newcastle upon Tyne, NE1 7RU UK

**Keywords:** Mitochondria, Egg donors, Germline alteration, Three genome embryo, UK legislation

## Abstract

Techniques for resolving some types of inherited mitochondrial diseases have recently been the subject of scientific research, ethical scrutiny, media coverage and regulatory initiatives in the UK. Building on research using eggs from a variety of providers, scientists hope to eradicate maternally transmitted mutations in mitochondrial DNA by transferring the nuclear DNA of a fertilised egg, created by an intending mother at risk of transmitting mitochondrial disease, and her male partner, into an enucleated egg provided by another woman. In this article we examine how egg providers for mitochondrial research and therapy have been represented in stakeholder debates. A systematic review of key documents and parliamentary debates shows that the balance of consideration tilts heavily towards therapeutic egg providers; research egg providers have been ignored and rendered invisible. However, mapping the various designations of therapeutic egg providers shows that their role is so heavily camouflaged that they have only an absent presence in discussions. We explore this puzzling ambivalence towards egg providers whose contributions are necessary to the success of current mitochondrial research and proposed therapies. We suggest that labels that diminish the contributions of egg providers serve certain governance objectives in managing possible future claims about, and by, therapeutic egg providers. We demonstrate that the social positioning of research egg providers is entangled within that of therapeutic egg providers which means that the former can also never receive their due recognition. This article contributes to the wider literature on the governance of new technological interventions.

## Introduction

In February 2015 the UK parliament agreed to pass into law regulations that change the Human Fertilisation and Embryology Act 1990 (HFE Act, as amended in 2008). These regulations will permit licensed clinics to use eggs and embryos that have been altered in specific ways, in treatment programmes for women at risk of having children affected by serious mitochondrial diseases. Mitochondrial disease takes many forms, some of which are caused by mutations in the small piece of DNA contained in each mitochondrion (mt-DNA).^1^ Scientists hope to be able to eradicate such maternally transmitted mt-DNA mutations by transferring the nuclear DNA of the intending parents into an enucleated egg provided by another woman; one which contains healthy mitochondria. This procedure eliminates the intending mother’s faulty mitochondria and will, scientists hope, result in a child free from the mt-DNA disease carried by the intending mother. Any children born as a result of such an intervention will therefore carry DNA from three sources, the two intending parents and the egg provider. Girls born of this procedure could go on to have children of their own who will carry the egg provider’s mitochondrial DNA. Such a change to the genetic makeup of these families has been described as germline modification (Tachibana et al. 2013) and remains illegal in most jurisdictions. Research on such modifications to eggs and embryos was permitted in the UK by an amendment to the HFE Act in 2001 but the potential use of such modified cells in a woman’s treatment was only made legal by the 2015 regulations.

These therapeutic developments are the culmination of at least 10 years’ research and the legislation was the culmination of several years of public consultations, ethical scrutiny and media coverage, all deemed necessary because of the germline modification involved. However, within these discussions there has been little acknowledgment of the women who provided eggs for the research that made these therapeutic applications possible in the first place. In addition, the roles of the egg providers in this therapeutic process, though acknowledged, have been labelled in various ways by various different groups and stakeholders contributing to the discussions. These labels are inadequate descriptors for the roles of those ‘donating’ (or as we prefer, providing) eggs for research, let alone for treatment, but we suggest that diminishing the role and importance of the contribution of these women, in both research and therapy, is a deliberate ‘strategy of persuasion’ (Haimes 2014) in the therapeutic context. These labels and their continuing implications for both the women concerned and the way the debates about mitochondrial research were and are shaped, are the subject of this article.

As we have noted elsewhere (Haimes et al. 2012; Haimes 2013), egg provision for research is a much-neglected area. While the field of ‘egg donation’ for the treatment of couples with fertility difficulties has received attention from various angles (Kenney and McGowan 2010; Gurtin et al. 2012; Pennings et al. 2014) there is far less empirical knowledge about egg provision for research, particularly from the perspectives of the women providing the eggs. The research that does exist suggests that the profiles of women volunteering to provide eggs, and the practices of their recruitment, are highly context-specific. For example, we investigated the values and experiences of IVF patients providing eggs for stem cell and somatic cell nuclear transfer research under an ‘egg sharing’ scheme in which they provide 50 % of their eggs in one IVF cycle and receive a £1500 discount on the fees for that cycle (Haimes and Taylor 2013; Haimes 2013). This study provided rare insights into the socio-ethical aspects of egg provision for research and argued that in the egg-sharing scheme the experiences of egg providers is heavily influenced by their social positioning within the IVF bio-economy. This then raises questions about the values and experiences, as well as about the social and medical profiles, of women who are not IVF patients but who volunteer to provide eggs for other forms of clinical research. We are currently seeking to fill that gap in knowledge by conducting research with women who are not IVF patients but who volunteer to provide eggs for research on mitochondrial disease; this empirical study provides the background to our analysis in this article.^2^

Our analysis contributes to the themes of this Special Issue by adding to our understanding of how the clinical labour (Cooper and Waldby 2014; Parry 2015) of egg providers for research and therapeutic purposes is, and is not, acknowledged and, when acknowledged, how it is framed in particular ways to achieve and establish certain understandings (and to exclude other understandings) of the social processes that underpin the scientific and clinical endeavours. Though the Special Issue has a particular interest in the provision of reproductive tissue for research, there is, in this article, an apparent preponderance of material about egg providers for treatment. This is far from irrelevant, however, as that predominance underscores the apparent absence of interest in, and concern for, egg providers for research; that in turn reflects the entanglement of research and therapeutic uses of provided eggs.

## The roles and contributions of women providing eggs for mitochondrial research

Through our current research we know that a young woman living in the North East of England hears through the local media, workplace websites, family or friends about the need for eggs for research. For example, she might hear a clinician on the radio talking about the children he treats who have conditions caused by the failure of what he describes as the ‘batteries’ in their cells and that the diseases are often passed from mother to child; the piece ends with an appeal for women to come forward to ‘donate’ eggs to assist this research. Possibly after some internet searching and discussion with family and friends, she decides to complete an online form indicating her interest in providing eggs and giving some initial personal information: name, date of birth, contact details and whether she wants to donate eggs for research or to help someone else have a child. This form is returned to the local fertility centre that works with scientists in recruiting egg providers and handling the medical interventions involved in providing eggs. If the young woman fits the initial filtering criteria for egg provision (age, geographical location) the clinic invites her to attend a meeting to get more information. If she decides to go ahead she is asked to sign a consent form to donate eggs and another for the medical interventions involved in providing eggs. She then undergoes medical screening, including a trans-vaginal ultrasound scan, being weighed and various blood tests to measure her ovarian reserve and, if indicated, a pregnancy test. Making the decision and giving consent to go ahead, and undergoing the medical screening, might happen on one visit to the clinic or over two visits.

She travels back to the clinic a couple of weeks later and is instructed on how to give herself injections. Over the next 4 weeks, once a day, she sterilises the tops of medication vials, draws up the dose, swabs a patch of her belly and injects herself. Over time, she learns that parts of her skin are more sensitive than others and that it is best not to use the same patch all the time because it gets sore. If she has stretch marks, she learns from experience that it hurts less to inject there. During this time she travels to at least two more appointments at the clinic, for scans to ensure she is not hyperstimulating dangerously. If all is going well, she is then taught how to dissolve and dilute a powdered hormone to the required dose. For a week she gives herself two injections of this each day and also attends the clinic for another scan. If the follicles are developing appropriately she is booked in for an egg collection procedure and she is given the final medication to trigger ovulation and told when she must inject that. She is told that she will need someone to accompany her on the egg collection day and to stay with her for 24 h until the effects of the sedative that she will receive for the procedure wear off. On the day of the egg collection, she is taken through to a small operating theatre where she is given the sedative. If she is one of the people resistant to the sedative, she will experience a painful egg collection rather than the ‘scratch’ and ‘discomfort’ she has been warned about. After her eggs have been collected she spends a couple of hours in a recovery room, at the end of which a member of clinic staff asks for a signature and gives her a cheque for £500. She can leave. After a few days of bad cramps she is back at work and able to reflect on her experiences.

This description is clearly a simplification; each element could be described in much greater detail to indicate the breadth and depth of this role (Baylis 2013; Waldby and Carroll 2012). Not only does the egg provider change her daily routines and give up time and money, she also draws upon the personal, familial, community and employment networks, within which she is embedded, to make her role as an egg provider possible, not least in organising or changing possible child care and or work arrangements. She asks for, or calls in, favours that will, in turn, have to be worked out or re-paid in the ongoing arithmetic of everyday social exchange.

We shall see below that this level of commitment and involvement in producing eggs for research is rarely acknowledged.^3^ Even less visible, though, are the experiences of those women who volunteer to be egg providers but who cannot be accepted for various reasons, such as a previously diagnosed or newly discovered medical condition or because of age, body mass index, or a range of other possible reasons. We do not have space here to discuss this aspect of the volunteering experience adequately but the implications of this experience for the women as individuals, and for the wider healthcare system, of how to manage such incidental findings, need consideration.

Now that legislation has been passed in the UK to allow this research to be translated into clinical applications additional women, who are willing to provide eggs to treat a couple at risk of passing on mitochondrial diseases, will need to be recruited. The above regime for providing eggs for research will need to be enhanced by more intensive health screening of, and the collection of more detailed family medical histories from, women volunteering to provide eggs for therapeutic purposes. In June 2015 the Human Fertilisation and Embryology Authority (HFEA), the body that will license clinics and regulate the therapeutic applications of this work, called for expert views to advise them on a number of requirements to implement this transition into therapeutic applications, including donor screening (http://www.hfea.gov.uk/6896.html). In September 2015 the HFEA stated that they would publish the ‘final processes, systems and guidance for regulating mitochondrial donation’ on October 29th 2015 (http://www.hfea.gov.uk/9862.html).

## What’s in a name? Diminishing the contributions of egg providers in mitochondrial research and therapy

### Troublesome terminology

Up to October 2015, any eggs provided can only be used for research aimed at developing and refining two therapeutic techniques: maternal spindle transfer (MST) or pronuclear transfer (PNT). Once the regulations permitting therapeutic applications come into force, newly provided eggs, with appropriate consent, will be used for therapeutic application.

This area of research and therapy is usually referred to as ‘mitochondrial donation’ or, ‘mitochondrial replacement’. For example, the HFEA, in the above-mentioned call for expert views, headed the relevant web page as ‘Mitochondrial Replacement’ and in the text refers repeatedly to ‘mitochondrial donation’ and to the ‘mitochondrial donor’. Those terms are misleading as they imply a direction of travel for the mitochondria that is in fact the opposite of what will actually occur. In the therapeutic application of PNT, an egg from the intending mother will be fertilised using sperm from the intending father. At the same time, an egg provided by a volunteer will be fertilised by the intending father’s sperm and that fertilised egg will be enucleated. The intending parents’ nuclear DNA, in the form of two pronuclei from their own fertilised egg, will then be transplanted into the enucleated egg which then acts as a ‘host’ to the transplanted nuclear DNA. MST will similarly involve the transfer of the intending mother’s nuclear DNA into an enucleated egg from the egg provider; that egg will then be fertilised by the intending father’s sperm. In other words, the egg provider’s mitochondria, whether in research or treatment contexts, are not moved anywhere. Therefore, rather than phrases such as ‘mitochondria donation/transfer/replacement’, a more accurate term would be ‘nuclear DNA hosting’, for both PNT and MST.

The term ‘mitochondrial donation’ also implies that the egg provider contributes only mitochondria; however, her ‘host’ egg contains not only mitochondria but also all the other cellular structures and chemicals required by the intending parents’ nuclear DNA to direct the egg to develop into an embryo. Therefore, in the therapeutic applications, not only will the transfer of material be in the opposite direction to that usually implied, but also the contribution of the volunteer’s host egg will be much more extensive than usually acknowledged.

Nonetheless, as we shall see, ‘mitochondrial donor’ and ‘mitochondrial donation’ are the terms most commonly used throughout the debates. Bredenoord and colleagues make a rare acknowledgment of the perpetuation of inaccurate terminology:

Although it would therefore be more precise to talk about ‘mtDNA replacement’ rather than ‘mtDNA modification’, we nevertheless use the term ‘modification’ as this is the term usually deployed in the literature ([Bibr CR3][Bibr CR4], p. 98).

However, ‘modification’ implies that the intending parents’ mitochondria will be altered by a (minor) contribution from the egg provider whereas in fact the intention of the intervention is to discard the intending parents’ mitochondria completely. We have also just described why ‘replacement’ is also problematic. These distinctions are important to note as the use of inaccurate terminology disguises the extent of the contribution of egg providers to the research that has enabled the science to get this far and will continue to obscure the contributions of egg providers to therapeutic applications.

### A brief history of UK mitochondrial science and policy

In the UK in 2005 the Newcastle Fertility Centre was granted a licence by the HFEA to conduct research on PNT. The researchers used abnormally fertilised eggs, from couples undergoing fertility treatment, for this research (Pincock 2005). In 2008 Parliament amended the HFE Act (1990) to include a provision for regulations to permit the use in treatment of eggs and embryos that had been modified to prevent the inheritance of serious mitochondrial diseases. Until such time as the regulations were enabled by Parliament, research could continue but the reconstructed eggs or embryos produced could not be used in treatment. In 2009 American researchers (Tachibana et al. 2009) described successful MST in non-human primates. In 2010 Newcastle researchers described the successful reconstruction of a human embryo using PNT and demonstrated minimal carry-over of mitochondria into the ‘donated’ egg (Craven et al. 2010). In February 2015 Parliament agreed to allow therapeutic applications.

We have reviewed the major U.K. documents from significant scientific, clinical, regulatory and bioethical stakeholders debating the permissibility of these therapeutic applications.

### The Nuffield Council on Bioethics (NCoB)

The independent UK bioethics organisation, the NCoB, convened a working party in 2012 to examine the issues around mitochondrial therapy, prompted by the view that Parliament would soon debate the approval of regulations. The drivers for changing the law were: (i) promising results from the Newcastle University mitochondrial researchers; (ii) growing pressure from patient groups; (iii) significant new investment in mitochondrial research by the Wellcome Trust; (iv) the Government asking the HFEA to seek the public’s views on the proposed new techniques and (v) the HFEA’s commissioning of a review of the science and its safety (HFEA 2011).

The NCoB report ‘sets out the ethical considerations arising from the possible use of such techniques for treatment in the future’ (NCoB 2012, xv). Although its focus is on treatment, rather than research, the report notes that,

4.146 One of the major barriers mentioned by scientists when assessing the potential for cell reconstruction techniques to become treatments is the fact that many more egg donors will need to be found to undertake the research required in order for the safety and efficacy of PNT and MST to be established, and if therapies are to be provided in future (2012, p. 83).

No attention was paid in the report, however, to the social and ethical aspects of recruiting and safeguarding those much needed egg providers for that ongoing research.

Throughout the report, the future therapeutic egg provider is most often referred to as a ‘mitochondrial donor’, though occasionally, as above, an ‘egg donor’. The egg providers are primarily discussed within three contexts: that of the social relationships formed by donation and assisted reproduction; the status of ‘mitochondrial donors’ in regulations, and the increased need for ‘egg donors’. For example,

5.13 The status of the mitochondrial donor in regulation should be carefully considered by Parliamentarians and regulators, particularly where this may bring with it implications for the perception of the potential social relationships engendered by the donation. While women undergoing a procedure in order to donate mitochondria would also be egg donors, in this instance their intention is solely that the relevant parts of their egg should be used in the reconstruction of another egg or embryo for the avoidance of genetic disease.

5.14 Accordingly, the Working Group does not take the view that the donor of mitochondria should be given the same status in all aspects of regulation as a reproductive egg or embryo donor (NCoB 2012, pp. 89–90).

This quotation is revealing in a number of ways: (i) as noted above it ignores and thus renders invisible the essential contributions of the current *research* egg providers; (ii) it neglects to consider the social and ethical aspects of therapeutic egg provision, especially from the providers’ perspectives; (iii) it focuses on the possible future familial relationships without even discussing the prior social relationships between clinicians and providers that underpin the ‘donation’ processes; (iv) it presumes to claim to know (without any evidence) the intentions of those future therapeutic egg providers; (v) it locates those intentions within the goal of avoiding genetic disease when that could be achieved in several ways without using MST and PNT (e.g. adoption); (vi) it invokes an existing debate about potential relationships in third-party assisted conception to advocate a different (lower?) status in the mitochondrial context, even though the egg provider’s role in mitochondrial therapy also results in third-party conception; (vii) it perpetuates the minimization of the egg provider’s role to that of ‘mitochondrial donor’ and designates the process as that of ‘donating mitochondria’ even though, as we have seen, the social and physiological contributions of the egg providers, and of the eggs, are much greater than those terms allow.

This report has been highly influential in setting the terms of the subsequent debates, in the UK Parliament and in the mass media, where similar tropes re-surface.

The wider bioethics literature on mitochondrial work rarely mentions the egg provider but instead tends to focus on the ethics of the procedure, the impact on any potential child, and on the ethics of germline modification (Bredenoord and Braude 2011; Bredenoord et al. 2011a, b). A rare exception is Baylis who, in considering the ethics of creating children with three genetic parents, also considers the harms to egg providers as one of four categories of objections to ‘mitochondrial replacement technology’ (2013, p. 531). She briefly documents the time and inconvenience involved in egg production and retrieval, noting that the American Society for Reproductive Medicine Ethics Committee estimate that egg providers undergo 56 h of interviews, screening, hormonal stimulation and egg retrieval (2013, p. 532). Our interviewees’ descriptions of the process suggest that this is an under-estimate, particularly when taking travel and other people’s time into account. Baylis expresses concern about the risks of coercion and exploitation of therapeutic egg providers and argues that the harm-benefit ratio is not in their favour.

### The Human Fertilisation and Embryology Authority

The HFEA was asked by the UK Government in 2011 to make an assessment of the science and safety of the proposed therapeutic techniques. Throughout the 45 pages of their experts’ report (HFEA 2011) there are references to ‘donor egg’, ‘donor zygote’ or ‘donor mtDNA’ but the ‘egg donor’ herself is referred to only twice: once in describing the sources of DNA in any resulting child and once in relation to matching the ‘egg donor’ and the ‘mother’ to overcome possible safety issues. Surprisingly the report does not even discuss the issues of the quality [other than the requirement for ‘normal mtDNA’ (2011:14)] or quantity of eggs needed.

Two further reviews (HFEA 2013a, 2014) continue in the same vein. A typical example of the way egg providers are referred to in the third report, which also cited the NCoB report (HFEA 2014, pp. 3–4) is,

At present the panel believes any risks are very low, but it recommends that if these techniques are used clinically, the latest evidence regarding how mtDNA haplotypes affect nuclear/mitochondrial interactions should be considered in order to inform *the donorselection process*. The panel also noted that in assessing this risk the treating clinician should be mindful of the *parallels* in natural reproduction and *current donor processes, such as organ transplantation or sperm and egg donation* (2014, p. 5 *emphasis added*).

In other words, the woman who will provide eggs that will enable another woman to have a ‘healthy’ child is seen, in this context, as doing something that *parallels* but is not the same as, established ‘egg donation’. Women who have already provided, or will provide, eggs for research purposes are not mentioned.

The HFEA were also asked by the Government to gauge public opinion on legalizing the proposed techniques. There is insufficient space here for a detailed analysis of the consultation materials but the final report (HFEA 2013b) refers to egg providers as ‘mitochondrial donors’ and always in the context of future egg providers for treatment; there is no reference to past, current or future research egg providers. For example,

…some stakeholders argued that current scientific evidence suggested that the role of mitochondria is limited to energy production and therefore, in their view, does not impact on a person’s physical characteristics. They felt that if *mitochondria donors* were treated on a par with gamete donors then this could have the perverse effect of de-valuing the status of gamete donation. And they felt that children born through mitochondria replacement may be curious to find out *details of their donor*—just as the recipient of a tissue donation might—but that curiosity is not enough to warrant providing identifying donor information (2013b, p. 24 *emphasis added*).

Once again there is an understatement of the contribution of the woman providing the egg and of the egg that has been provided, accompanied by a down-playing of the crucial role of the ‘donor’ in assisting the birth of a child free from mitochondrial disease. Additionally, there is a set of non-evidenced claims of what any resultant child might want to know, and why they might want to know it, about the egg provider and a dismissal, again without evidence, of that as mere ‘curiosity’. They do not explain why they think there could be problems from identifying egg providers, nor do they defend their conclusion or justify whether that is ‘enough to warrant’ their decision.

Another example of the ways in which egg providers are viewed in limited, instrumental ways and as a mere means to an end, rather than as agents in their own right, can be found in an HFEA-produced video, that was part of their public consultation exercise. In the video, in which mitochondrial disease is explained, the mitochondria are described as being like power plants, providing energy for cells; the intending parents are represented by cartoon outlines of a man and a woman but the egg provider (‘donor’) is represented by a cartoon drawing of a power station (https://vimeo.com/45389280).

### The Department of Health

In late 2014, the UK Department of Health (DH) opened a public consultation on the draft legislation that was to be laid before Parliament. A consultation document was prepared by the DH Health Science and Bioethics division to explain the intended effects of the legislation in an accessible manner. In this document the egg provider was referred to as an ‘egg donor’ in the context of describing ‘three person IVF’ (DH 2014, p. 13) and as a ‘mitochondrial donor’ when describing how the legislation will ensure that she ‘does not have the status as a generic (*sic*) gamete donor’ (2014, p. 16) and ‘should be treated more like [an] organ donor’ (2014, p. 20). The egg provider is a ‘mitochondrial donor’ or simply ‘the donor’ when reasons are laid out for her remaining anonymous to any child born as a result of the proposed techniques. The consultation document mentions that research on the proposed techniques is ongoing, but makes no reference to where or how the eggs for that research are obtained. The consultation document and the draft legislation focus on future treatment and make no reference to providers of eggs for research. The draft regulations echo these same limited representations of egg providers.

### Parliamentary debates

Newspapers and other mass media outlets have displayed a lively interest in this field since 2013 and have been shaped by, and have helped to shape, the idioms of understandings of other stakeholders. Television, radio and newspaper journalists have contributed to the debate by focusing either on the scientific techniques and or on the families with mitochondrial diseases who might be helped by the proposed interventions. Coverage and discussion of egg providers is limited to their being contributors to ‘three-parent babies’. The pervasiveness and influence of this phrase can be seen in the Parliamentary debates, where it appears in the titles of the Hansard reports of debates and the spoken contributions of most participants.^4^

A number of debates of different types have taken place in Parliament since the 2010 publication by Newcastle researchers (Craven et al. 2010). Two debates took place in Westminster Hall in 2013 and 2014, two in the House of Commons, in 2014 and 2015, and one in the House of Lords in late February 2015.

Westminster Hall debates are occasions in which backbench Members of Parliament (MPs) air subjects of concern to them or their constituents, seek updates on issues from Government Ministers, and influence other Parliamentary procedures; hence these debates inform the legislative process. Egg provision was mentioned in the first Westminster Hall debate (*Mitochondrial Disease*) (Hansard 2013) only in the context of providing eggs for the proposed treatments, not research. The women who will provide eggs were referred to as ‘healthy individuals’, ‘donors’ and ‘mitochondria donors’ and were discussed in relation to their lack of genetic influence on future children and in relation to their proposed anonymity.

The second Westminster Hall debate was brought by Rees-Mogg MP who stated that his views are ‘strongly influenced by the Catholic Church concerning the dignity of the human person’ (Hansard 2014a, Col 167WH). He refers to women who might provide eggs for MST, in the following way,

…the embryo has three parents: the spindle mother, the egg donor mother and the father. Genetic parenthood is complete in the case of the father but fragmented in the case of the two mothers^5^ (2014a, Col 164WH).

‘Donor’ and ‘mitochondrial donor’ were the other terms used throughout the debate and again the egg providers were spoken of only in terms of the instrumental use to which parts of their bodies were to be put. At no point in the debate was there any mention of the contributions made by, or concern for the wellbeing of, the women who provide eggs to facilitate the *research* on the proposed interventions.

Another route for concerned MPs to bring issues to the attention of the House of Commons is via a debate arranged through the Backbench Business Committee. One such debate was granted to Bruce MP, ‘*Mitochondrial Replacement* (*Public Safety*)’ (Hansard 2014b). This resulted in a motion being agreed to call on Government to delay bringing forward legislation to allow therapeutic applications on the grounds of public safety. ‘Donors’ were mostly mentioned in this debate in the contexts of their providing only a small number of genes to the child.^6^ However, unusually, one MP raised the risks of ovarian hyperstimulation syndrome (OHSS) for egg providers:

Glindon: … No in-depth questions about the physical health of women donating eggs were addressed in the HFEA’s own briefings. Will the Minister assure the House that she will take the matter back to her Department for consideration? In the light of the safety concerns we have heard today associated with the proposed techniques, such action would appear to be urgent, for the sake of the vulnerable women involved (2014b, Col 97).

This statement conflates two different aspects of safety (that of providing eggs and that of the transfer of DNA from the intending parents into an enucleated egg) so it is unclear which women (intending mothers or egg providers) are ‘vulnerable’, or indeed whether all women were so regarded.

The two debates in February 2015 were the centrepieces of the legislative process. In the House of Commons debate on February 3rd, 2015 on the ‘*Draft Human Fertilisation and Embryology* (*Mitochondrial Donation*) *Regulations 2015*’ egg providers were referred to as ‘healthy donor’ or ‘donor’, and at no point was the process of egg provision discussed (Hansard 2015a). In the House of Lords debate on February 24th 2015 the only reference to women providing eggs (‘donors’) was in the context of their safety and the possibility of OHSS. This was raised by both Lord Alton and Baroness O’Loan who both opposed the introduction of the proposed techniques, because of their religious (Catholic) beliefs (Hansard 2015b). The votes following these debates started the process by which the HFE Act was altered to permit the licensing of clinics to carry out the proposed techniques in therapy.

## The absent presence of the therapeutic egg provider in the ‘three-parent’ baby debates

It is clear from the analysis of the above documents that the egg provider receives inadequate attention in discussions, given her contributions to mitochondrial research so far and her likely future contributions to therapeutic applications. However, it is worth returning briefly to the area in which she does have an explicit presence: that is, in the phrasing of ‘three-parent IVF/embryo/baby’. The ‘three parent’ terminology is so prevalent that not only does it feature heavily in media coverage of these issues, it even featured in the title of the March 2014 Westminster Hall debate, ‘*Mitochondrial Transfer* (*Three*-*parent Children*)’ (Hansard 2014a, col 164WH). In this usage, ‘parent’ appears to be used as shorthand for genetic contributor, though this conflation can of course be challenged on the grounds that ‘parent’ has a much wider social meaning, relating to raising a child. However, and curiously, it has been challenged, particularly by those in favour of mitochondrial work (Baylis 2013), but on different grounds; that is, on the *amount* and *type* of genetic contribution from the egg provider. For example Onwurah, a Newcastle MP argued,

I want to focus on the so-called three parents issue. The embryo would carry just 13 out of 23,000, or 0.056 %, of the genetic material from the mitochondrial donor. …it is not the nuclear DNA, so the child’s appearance, personality and other features are not affected (Hansard 2014b, cols 112–113).

Similarly, the Chief Medical Officer, Dame Sally Davies, wrote:

It is important to remember that mitochondrial DNA represents less than 0.054 per cent of the total DNA, and is not part of the nuclear DNA, which determines our personal characteristics and traits such as personality, hair and eye colour (Davies 2015).

The clear implication is that mitochondrial DNA is relatively unimportant. In bioethics too, this idea apparently has some currency; Wilkinson (2014) argues,

For while the children created will be genetically linked to the donors, it’s far from clear that that link is sufficient to constitute parenthood. Only around 0.1 % of our genes are contained in mitochondria (with the other 99.9 % being in the cellular nuclei) and so the mitochondrial donor only provides a tiny minority of the child’s genetic material.

However, as we have seen, the provided egg contributes much more: not only this DNA but also the initial environment for effective embryo development. Also, however numerically small the genetic contribution, it is nonetheless the crucial amount; so crucial that legislation has been enacted to allow these techniques to go ahead, because it is precisely this DNA that will (if the IVF is successful) lead to a child free from mitochondrial disease. This is likely to have a profound influence on that child and the development of his/her identity and personality. Hens et al. (2015), reporting a study of professionals’ views on the use of mitochondrial therapy, mention that the contributions of egg providers were an area of debate amongst their interviewees. Some ‘were not convinced that the role of the mtDNA donor was trivial’ (2015, p. 1259); the authors suggest that ‘the status of the donor of the mitochondria may evolve as more becomes known about the role of mtDNA’ (2015, p. 1261).

Baylis is clear that this attempt to minimise the genetic contribution arises from a wish to ‘downplay the relevance of “third-party” mitochondria in an individual’s genetic makeup’ (2013, p. 532) and in response argues the same point as we have done, that the mitochondria from the egg provider, however slight numerically, will profoundly affect the child’s identity. She therefore defends the disputed terminology arguing that ‘any child born following the mitochondrial replacement would have three genetic parents’ (2013, p. 532). In concurring with the view that the personal, social and psychological impact of this small amount of DNA will be profound, we would nonetheless argue that the more appropriate terminology would be ‘three genome IVF/embryo/baby’. The question of genetic, social, legal and psychological parentage is far more complex (Haimes 1990; Strathern 2005; Widdows and Baylis this issue) and requires a distinct debate; it cannot be hidden behind, nor resolved by, claims based on the quantification of DNA. It is also a debate to which actual and potential egg providers can make a vital contribution, even though so far they have been excluded from so doing. This exclusion is likely to continue into the future: even Hens and colleagues, when suggesting there should be further exploration of the opinions of ‘people affected by mitochondrial diseases and of the general public’ (2015, p. 1261), neglect to suggest exploring the views and experiences of egg providers.

## Discussion

We have shown in the above review how key documents and institutions have framed egg providers in mitochondrial research and therapy. To summarise: egg providers for *research* are crucial to getting to the stage of mitochondrial therapeutic applications but are hardly mentioned in any of the debates or consultations that have taken place. Potential egg providers for *therapeutic* applications are mentioned but only briefly and only in order to minimise their contributions. This results in an anomalous characterisation of egg providers in this field, in which they are both crucial but also invisible. Egg providers for research are rendered invisible by being ignored; egg providers for treatment have a presence in debates but only in terms that diminish or negate their contributions, hence our reference to them as having an ‘absent presence’. Distancing and reductionist labels work as a mechanism to ensure that there is little acknowledgment of research providers and a diminution of the role of therapeutic providers. What does this suggest about the social and bioethical positioning of egg providers in mitochondrial and other biotechnological and clinical research?

The terminology and labels applied to the role of egg providers in these discussions comprise a ‘lexicon of persuasion’ (Haimes 2014; Fernandez 1986) that conveys a sense of the essential unimportance, other than in merely technical terms, of the egg providers and of their eggs; they are simply a means to an end. It could be claimed that this is the result of a certain carelessness in the research context as egg providers, and providers of other bodily material in other research contexts, are routinely rendered invisible (Haimes and Luce 2006; Haimes and Taylor 2011). For example, it is noticeable that readily available photographs on research websites that describe and illustrate the ‘egg harvesting’ process not infrequently label all elements in the photograph, including the surgical instruments used and the photographer’s name, but fail even to acknowledge the body from which the eggs are being collected, let alone the woman whose body that is. This creates an ‘absent presence’ of providers (Haimes 2015) in which eggs are ‘harvested’ but women hidden; the individual woman is reduced to a disembodied egg. Such carelessness is no longer excusable, however, given how much has now been published about the importance of egg providers, and the providers of other bodily materials, for research (NCoB 2011).

Such socio-linguistic practices can be construed as a deliberate strategy in the therapeutic context. The persistent (i) failure to acknowledge the fully rounded social, psychological, familial, emotional and physical contributions of the egg provider; (ii) emphasis on the very small amount of genetic material being provided; (iii) obfuscation of the physiological contribution of the whole egg environment; (iv) denial of the impact of the third genome in the resultant child’s hoped-for mitochondrial disease-free future and personality development, have a collective (and one can assume, intended, given that persistence) impact. Together they constitute a *strategy* of persuasion (Haimes 2014): that is, by enrolling particular characterisations of the contributions of the women who provide eggs, the scientific, clinical and bioethics participants in the debates frame instructions to policymaking and public stakeholders to hear the terms of debate in a particular way. These labels persuade audiences to diminish the role of the egg provider and the egg provided, while simultaneously downplaying the significance of the procedure. This reduces the egg providers and eggs to ‘just tissue’, ‘only mitochondria’, ‘only 13 genes’. This then permits the debate to be framed in terms of pre-existing tissue and organ donation in which practices such as the anonymity of tissue and organ providers is already established as the apparently accepted norm (Sharp 2006). We are perhaps also witnessing a ‘strategy of ignorance’ (developed from Hoeyer et al.’s ‘deliberate ignorance’, 2015) where stakeholders affect not to know the full role and contributions of egg providers in order to persuade others that this is not significant in the overall picture. In the mitochondrial field, these combined strategies helped to smooth the path to legalisation in the UK in 2015.

Crucially, these strategies also act, as we have seen, as counters to the idea of egg providers in mitochondrial therapeutic applications being seen as any kind of parent to the child conceived through these treatments. And yet, given that these interventions are all directed at enabling the intending parents to be genetic parents (otherwise they could use adoption, surrogacy and/or a whole egg provided by another woman instead) this creates a central irony in mitochondrial therapy: that, in order to have a genetically related child, the intending parents have to involve a third genetic contributor, the egg provider. It is the crucial involvement of a third genome that makes the goal of being a genetic parent possible at all. This suggests that the role of the egg provider should be celebrated.

However, the social and linguistic practices and arrangements identified in this article act not only to undervalue the contributions of the egg provider to both research and therapeutic purposes but also to camouflage those contributions and, by association, the women making those contributions. It is as though, provocatively, the egg provider, particularly in the therapeutic context, were ‘the other woman’ in an altogether different sense; a shameful secret to be hidden and lied about, rather than respected and celebrated, whose exposure causes disruption and disgrace, not only to her but also to those associated with her. These associations are perhaps reinforced by the intending parents using an egg from this ‘other woman’ that has been fertilized by the male partner’s sperm.

The reluctance of scientists, clinicians and policymakers to engage systematically and in-depth with the involvement of egg providers in mitochondrial therapy reflects a long history of uncertainty about the management of biological relationships in assisted conception (Haimes 1990; Thorn 2015; Widdows and Baylis this special issue). The obfuscation of the role of egg providers in mitochondrial therapeutic applications is merely one instance in a long line of such strategies. This reveals the deep ambivalence amongst scientists, clinicians and policymakers towards the contributions of third parties in assisted conception: they need the assistance of gamete providers but also fear what their involvement will bring in terms of complex future social relationships, so they act in ways that disguise the centrality of gamete donors to the therapeutic process. However, trying to downplay or even disguise the roles and contributions of egg providers does not resolve any potentially difficult issues; it simply brushes them under the carpet.

The evidence from discussions with interviewees in our current project on egg providers for mitochondrial research about the possibility of whether they would also provide eggs for therapeutic purposes,^7^ though not fully analysed yet, suggests that women contemplating giving eggs for therapeutic purposes are highly conscious of the wider familial and social ramifications of helping another woman to have a child. They are also very alert to the distinctions between contributing to a child's conception and having a parenting role in that child’s upbringing; they are aware of longer-term issues that might arise if, when that child reaches adulthood, s/he expresses an interest in the egg provider’s role in their conception. These interviewees approach such issues with consideration, compassion and subtlety.

It is also noticeable that the very few times that the egg provider was mentioned in her own right in the Parliamentary debates, it was by those opposing the full range of mitochondrial interventions. The risks that the egg provider might encounter were merely items on a longer list of objections to mitochondrial research and germline modifications in therapeutic applications. This is reminiscent of the UK Parliamentary debates on the creation of hybrid animal-human embryos in 2008; on one of the few occasions where attention was paid to egg providers they were invoked as encountering possible risks and harms by those in support of hybrid embryos, as the grounds for advancing their case to permit the use of cow eggs instead (for example, Hansard 2008, Col 1101). None of those dangers was raised in the mitochondrial debates other than by those few voices opposing *any* intervention. In other words, in both cases, the egg providers are used as a means to scientific and political ends, rather than as ends in their own right.

In the mitochondrial field (and possibly all fields depending on third party gametes and embryos) the neglect of *research* egg providers takes on an added significance given the strategic downplaying of the role of *therapeutic* egg providers. Since that downplaying is directed towards pre-empting arguments about, and undermining possible future claims to, parenting status by the provider, it is even less likely that the contributions of *research* egg providers will ever be acknowledged, let alone celebrated. It would be difficult to do this and then still fail to acknowledge the even greater contribution (particularly to the resultant child) of the therapeutic egg provider. In other words, the strategy of persuasion on the role of therapeutic egg providers is underpinned and reinforced by what can now be seen as the strategic neglect of the contributions of research egg providers.

From a bioethics point of view, this of course raises questions about dignity. Though a notoriously slippery concept (Hayry 2004; Mattson and Clark 2011) it derives, in part at least, from receiving full due respect for one’s contributions to any endeavour. Sayer, for example, argues that dignity is associated with, amongst other conditions, ‘respect, pride, recognition, worth and standing or status’ and is negatively related to ‘shame, stigma…lack of recognition or trust’ (2011, p. 192). A relational notion of dignity suggests ‘the idea of dignity as something crucial yet fragile which depends on how people act and how others treat them; indeed, as if it depended on its being recognised’ (2011, p. 192). He concludes that ‘Dignity and respect are crucial for people’s well-being and people who are denied respect tend to care deeply about this. Yet it is also not much to ask for.’ (2011, p. 215). The avoidance of these more complex bioethical and sociological issues in the mitochondrial field reflects the tendency noted elsewhere of rendering complex socio-ethical challenges into merely technical issues (Dreyfus and Rabinow 1982): in the mitochondrial field, the focus is on how to get the ‘batteries’ or the ‘power station’ to work effectively, rather than according recognition to the women whose contributions are vital to this process.

This use of labels to downplay, and the failure to respect, the contributions, and indeed dignity, of egg providers is part of wider practices within bio-politics and governance more generally. Hacking, for example, has ‘long been interested in classifications of people’ and in ‘the ways in which a new scientific classification may bring into being a new kind of person, conceived of and experienced as a way to be a person’ (Hacking 2006, p. 2). These are not mere labels but have significance for the ways in which those categories of persons are then managed through structures of governance such as scientific, clinical and policy guidelines and legislation. Just as important though is what Hacking calls the ‘looping effect’; that is, how these classifications ‘affect the people classified, and how the effects on the people in turn change the classifications’ (2006, p. 2). On a practical point, will actual and potential egg providers who hear themselves being designated as mere ‘mitochondrial donors’ or simply as ‘tissue donors’, come to think that their contributions are not so important after all and consequently decline to undergo the complex, time consuming and arduous processes of providing eggs? We do not yet know how these processes of ‘making up people’ (2002, p. 99) (see also Campbell and Stark 2015) and managing them in mitochondrial research and therapy will affect those providing eggs but it is an important area to continue to research, if only to challenge the dominant categories that are emerging, in the UK debates at least. As McCallum (2014) argues, we need to take seriously ‘the governing actions of science itself as the determinant of normal life …’ (2014, p. 468).

A frequent rebuttal to the sort of analysis presented in this article argues for the need to focus on the bigger, therapeutic, picture and on the contributions of the central clinical research to those suffering from mitochondrial and other diseases (Thompson 2015). However, sometimes the bigger picture is not as big as claimed (Baylis 2013). Also, attention still needs to be paid to detailed, situated, practices in order to highlight the essential contributions of others to this bigger picture because, not only is the devil in the detail, but so too is the generosity and courage of research and therapeutic egg providers. It is only by fully recognising and celebrating this, that we can also fully celebrate the science and its successes.

## Footnotes

1 The majority of DNA in humans is contained in each cell’s nucleus, however, mitochondria have their own DNA. Mitochondrial DNA is transmitted from one generation to the next only through the maternal line; mitochondria present in the egg provide the basis for all mitochondria in the offspring.

2 Views of women volunteering to provide eggs for mitochondrial research. Wellcome Trust award number WT1022609.

3 We are not suggesting at any point in this article that egg providers are neglected in any way in terms of clinical care or management.

4 Examples of headlines include ‘World’s first ‘three parent’ babies could be born in the UK’, The Guardian, December 18th, 2014; ‘MPs clear the way for three-parent babies’, February 4th, 2015, Mail Online.

5 This use of ‘mother’ is problematic in another sense to that implied by Rees-Mogg, since it suggests that these therapeutic interventions will always be successful, when the current IVF success rate for women of all ages is around 26 % (HFEA 2013c); he is not unusual in making this error.

6 For example Rees-Mogg (Hansard 01/09/14, col 98) argued that although only 0.1 % of the child’s DNA changes, this is still a change in the child’s identity; see also columns 101, 107 and 121 for other Parliamentarians making similar arguments.

7 See footnote 1 for details.
